# P51 Enrichment of drug resistant *Klebsiella pneumoniae* in the gut following antibiotics alters the colonic immune landscape

**DOI:** 10.1093/jacamr/dlaf230.058

**Published:** 2025-12-04

**Authors:** Ffion Hammond, Claire Pearson, Elizete Batista Neves Araujo, Fiona Powrie

**Affiliations:** Kennedy Institute of Rheumatology, University of Oxford, Oxford, UK; Kennedy Institute of Rheumatology, University of Oxford, Oxford, UK; Kennedy Institute of Rheumatology, University of Oxford, Oxford, UK; Kennedy Institute of Rheumatology, University of Oxford, Oxford, UK

## Abstract

**Background:**

*Klebsiella pneumoniae* (*Kp)* commonly colonizes mucosal surfaces, with high hospital-acquired colonization rates. Gastrointestinal colonization with antimicrobial resistant (AMR)-*Kp* increases patient risk of subsequent infection, via unclear mechanisms. We hypothesize that antibiotic treatment facilitates AMR-*Kp* expansion, leading to altered immune landscapes (colon and secondary sites), creating vulnerability to subsequent infections.

**Methods:**

To investigate how AMR-*Kp* colonization influences the local immune response and native microbiome, germ-free (GF), Gnotobiotic Oligo-MM^12^ mice (representative minimal microbiome of 12 physiologically relevant bacteria, MM12), or specific pathogen free (SPF) mice were orally gavaged with AMR-*Kp* strain KP35. Gastrointestinal colonization was monitored through stool cfu.

**Results:**

KP35 poorly colonizes SPF mice but does stably colonize MM12 and GF animals. This colonization does not result in overt histopathology or inflammation of the colon. Antibiotic treatment of MM12 and SPF mice results in KP35 expansion in the gastrointestinal tract, comparable to levels observed in GF mice. KP35 expansion is accompanied by increased colonic neutrophils and relevant recruitment markers (determined by flow cytometry and qPCR respectively) for the duration of antibiotic treatment. When antibiotic treatment is stopped, KP35 cfu drops back below detectable limits. KP35 expansion is also observed in the lung following antibiotics in both MM12 and SPF models, and preliminary studies suggest AMR-*Kp* colonization combined with antibiotic treatment can influence subsequent lung infection. Therefore, AMR-*Kp* gastrointestinal colonization is influenced by microbiome complexity, which is disrupted by antibiotic treatment, leading to an expansion of AMR-*Kp* and recruitment of neutrophils to the colon. AMR-*Kp* expansion within the gut may not be overtly pathogenic but may generate a more ‘primed’ environment and influence mucosal barrier integrity making the host more vulnerable to secondary infections.

**Conclusions:**

Understanding microbe or host factors that may prevent or reduce AMR-*Kp* colonization and antibiotic-induced enteric blooms could provide new therapeutic avenues for opportunistic AMR infections.

